# An epiQTL underlying asexual seed formation in Arabidopsis

**DOI:** 10.1007/s00497-024-00504-y

**Published:** 2024-06-05

**Authors:** Rishabh Pankaj, Shiana Shoejaeyfar, Duarte D. Figueiredo

**Affiliations:** 1 Max Planck Institute of Molecular Plant Physiology, Potsdam Science Park, Am Mühlenberg 1, 14476 Potsdam, Germany; 2 Business Academy Aarhus, 8260 Viby J, Denmark

**Keywords:** Endosperm, Apomixis, DNA methylation, Auxin, Seed development, QTL

## Abstract

**Key message:**

The DNA methylation status at an epigenetic quantitative trait locus in the Arabidopsis chromosome 2 is linked to the formation of apomictic-like endosperms.

**Abstract:**

Seed development in most angiosperms is coupled to fertilization of the maternal gametes by two sperm cells. However, apomictic species can reproduce asexually via seeds. This trait is of great agricultural interest, as it would fix complex genotypes and allow for pollen-independent seed production. However, engineering full apomixis requires three independent processes: apomeiosis, parthenogenesis and autonomous endosperm development. While the first two have been successfully engineered in some crops, the formation of autonomous endosperms remains a challenge. Although it is known that this trait is under epigenetic control, such as of DNA methylation, the underlying mechanisms remain mostly undiscovered. Here, using epigenetic recombinant inbred lines, we identified an epigenetic quantitative trait locus in the Arabidopsis chromosome 2, which correlates with permissiveness for the formation of asexual seeds: hypomethylation at this genomic region allows the formation of larger autonomous endosperms. Importantly, the methylation at this locus only correlates with asexual seed size, and not to the size of sexual seeds or that of other organs. With this, we aim to show that screening for epialleles is a promising strategy to uncover loci underlying relevant traits and could pave the way to identifying genes necessary for the engineering of apomixis.

**Supplementary Information:**

The online version contains supplementary material available at 10.1007/s00497-024-00504-y.

## Introduction

While most angiosperm ovules require fertilization in order for seeds to form, ovules of apomictic species can bypass fertilization and produce asexual seeds. This trait is genetically complex and involves three independent steps: apomeiosis, parthenogenesis and autonomous endosperm development (Hand and Koltunow 2014). The introduction of apomixis into crops is highly desirable, but although there have been advances in engineering the first two components of apomixis in sexual species (Marimuthu et al. 2011; Khanday et al. 2019; Wang et al. 2019; Chen et al. 2022; Vernet et al. 2022), engineering autonomous endosperms remains an outstanding challenge. Importantly, autonomous endosperms are produced in Arabidopsis mutants lacking the gametophytic fertilization independent seed-polycomb repressive complex 2 (FIS-PRC2) or via exogenous or ectopic auxin (Chaudhury et al. 1997; Kiyosue et al. 1999; Ohad et al. 1999; Roszak and Köhler 2011; Figueiredo et al. 2015). However, those autonomous seeds are short-lived, meaning that additional pathways are necessary to form viable autonomous endosperms. In addition to the FIS-PRC2-controlled pathways, DNA methylation (mC) also regulates autonomous seed formation (Vinkenoog et al. 2000; Schmidt et al. 2013), but the underlying mechanisms remain undiscovered. We hypothesized that the mC status of specific loci, rather than global hypomethylation, should control the size of autonomous seeds. To test this, we used a collection of Arabidopsis epigenetic recombinant inbred lines (epiRILs) (Johannes et al. 2009) and screened their capacity to form autonomous seeds. We thus identified an epigenetic quantitative trait locus (epiQTL) linked to autonomous seed formation. Importantly, the mC status at the epiQTL specifically affects the size of asexual seeds, but not that of their sexual counterparts. We thus show that screening epigenetic variation is a useful strategy to uncover alleles underlying important agronomic traits, such as apomixis.

## Results and discussion

**Fig. 1:**
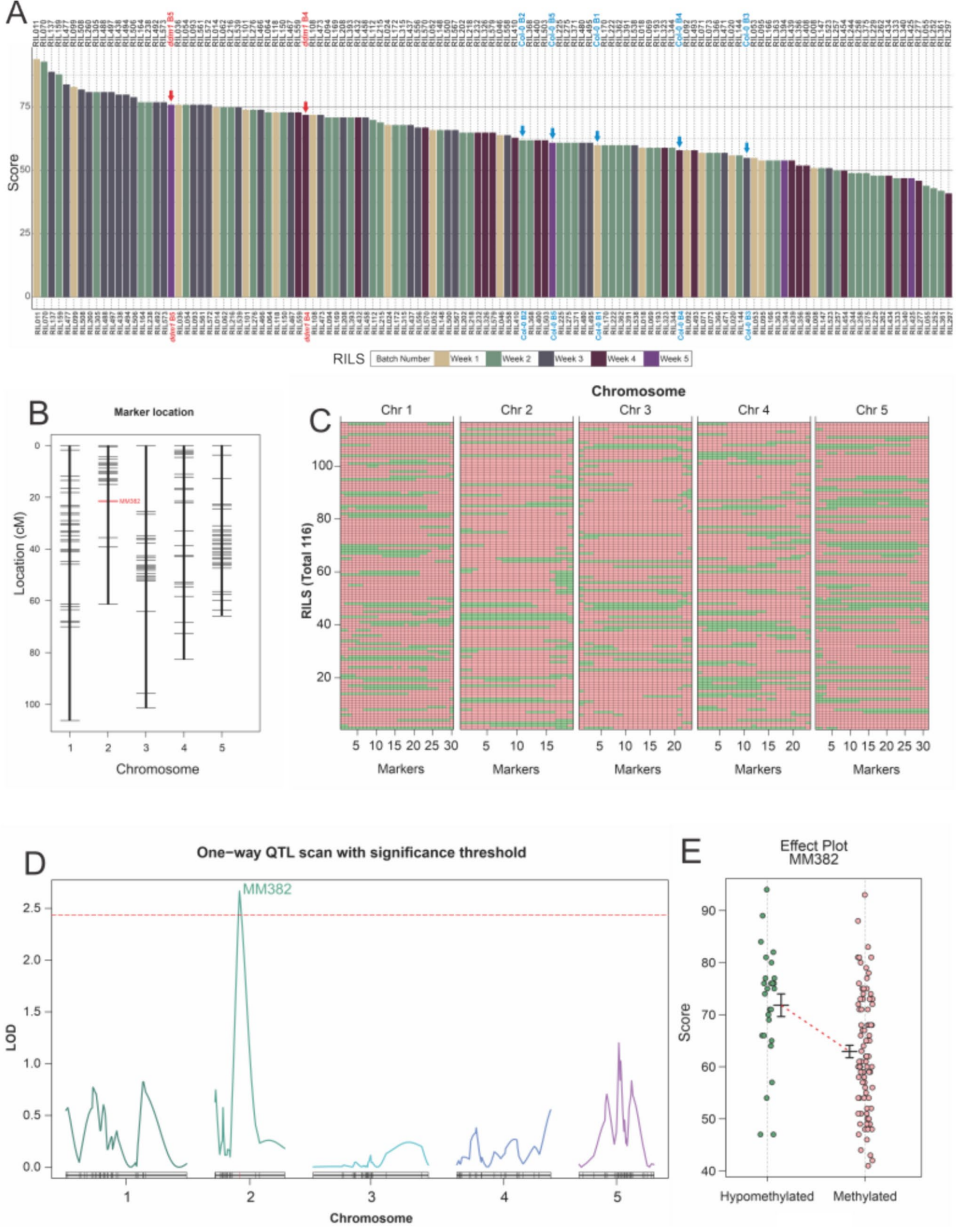
**A** Autonomous seed scores after 100 µM auxin treatment, as determined in 116 epiRILs, two *ddm1-2* lines (red label/arrow) and five WT lines (Col-0; blue label/arrow). The bar color code indicates which lines belong to which batch of plants. **B** Distribution of molecular markers used for epigenotyping distributed along the five Arabidopsis chromosomes. Marker MM382 is highlighted in red. **C** Distribution of methylated (pink) and hypomethylated regions (green) in the 116 individual epiRILs. **D** One-way QTL scan of epiRILs based on scores generated after auxin treatment of epiRILs. The red dashed line represents the LOD significance threshold. Peak DMR marker (MM382) with the highest LOD scores is shown on top. **E** Distribution of effect plot between peak marker haplotype MM382 (hypomethylated vs. methylated) and autonomous seed size scores

First, we confirmed that DNA hypomethylation promotes autonomous seed formation: exogenous auxin treatments of the mC mutants *ddm1* and *met1* results in larger autonomous seeds than in the wild type (WT; Fig. S1). We then repeated this experiment in a collection of epigenotyped epiRILs (Colome-Tatche et al. 2012) and measured autonomous seed size 3 days after the treatments (3 DAT; Fig. S2). We attributed a score to each line, which we used as a trait for epiQTL mapping (Fig. 1A). A linkage map of stably inherited DMR markers was used for interval mapping (Fig. 1B, C), and this revealed one statistically significant epiQTL on chromosome 2, associated with molecular marker 382 (MM382; Fig. 1D). The mC status at the epiQTL, as determined for MM382, showed a positive correlation with the autonomous seed size score (Fig. 1E): epiRILs demethylated in MM382 showed a 14% score increase, compared to those where this locus is methylated. Although based on outliers on the effect plot, we cannot rule out the presence of additional weaker epiQTLs contributing to autonomous seed formation that were not detected in our analysis. The epiQTL spanned a region of almost 2.8 Mb in Chr. 2: between positions 9,652,952 to 12,449,489 in the Arabidopsis TAIR10 genome. We then searched whether genes known to be involved in endosperm development were located within this region. Of all genes encoding FIS-PRC2 members, only *FIS2* is in chromosome 2, but is located outside of the QTL region. Regarding other PRC2 genes, only *CURLY LEAF* (AT2G23380), which encodes a component of sporophytic PRC2s, is within the QTL interval. Loss of sporophytic PRC2 function does lead to larger autonomous seeds, and to autonomous development of the seed coat (Roszak and Köhler 2011; Pankaj et al. 2023). Therefore, to test if the size of autonomous seeds was linked to ovule size, and namely to autonomous seed coat formation, we measured unfertilized ovules for the top 6 epiRILs with the largest and the top 4 epiRILs with the smallest autonomous seeds. There was no visible pattern in ovule size, when compared to the performance of each epiRIL in producing autonomous seeds (Fig. S1). This means that autonomous seed size after auxin treatments is not dependent on the initial size of the ovules, or on the autonomous development of their seed coat, but on factors driving their growth following the auxin treatments. We did not find other known regulators of seed growth within the epiQTL region.

We then tested if mC status at MM382 correlated with other developmental phenotypes. For this, we selected the seven epiRILs that produced the largest autonomous seeds and were hypomethylated at MM382. First, we confirmed that those epiRILs were indeed more permissive to the formation of autonomous seeds. Indeed, six out of seven epiRILs demethylated in MM382 produced autonomous seeds that were significantly larger than the WT (Fig. S1). However, we did not see an increased prevalence of autonomous endosperms in the RILs (Fig. S1). These observations indicate that DNA methylation at this marker is linked to autonomous seed size and not to initiation of autonomous endosperm.

It is well established that *ddm1* mutants are late flowering (Kakutani 1997), which we confirmed (Fig. S3). However, none of the selected epiRILs was as delayed as *ddm1* when it comes to bolting time, suggesting the loci responsible for bolting and those responsible for autonomous seed size are different. Then, we tested if the mC status at MM382 influenced the size of other organs, or if its effect is specific to autonomous seeds. We scored plant height, and root and leaf size. In the case of plant height and root growth, the epiRILs did not follow any visible pattern, when compared to their performance in producing autonomous seeds (Fig. S3). These observations suggest that hypomethylation at MM382 does not lead to general increased organ growth. This is in line with previous observations where MM382 did not overlap with epiQTLs identified for flowering time and root length (Cortijo et al. 2014). The only exception that we found was for leaf size (Fig. S4), where half of the tested RILs had larger leaves when compared to the WT control. This fits with previous observations where an epiQTL responsible for leaf growth was associated with MM382 (Kooke et al. 2015).

We then hypothesized that the increased autonomous seed phenotype linked with marker MM382 could be due to increased sensitivity to auxin. In that case, epiRILs demethylated at MM382 should respond more strongly to auxin, when compared to WT. We tested this by scoring root elongation in auxin-supplemented medium (Fig. S3). Interestingly, while the WT produced shorter roots in the presence of auxin, this was not so for *ddm1*, suggesting that global genome hypomethylation in fact leads to reduced sensitivity to auxin. Regarding the epiRILs, root length again did not show any visible pattern under auxin treatments. This indicates the loci controlling autonomous seed size are not linked to responsiveness to auxin.

**Fig. 2:**
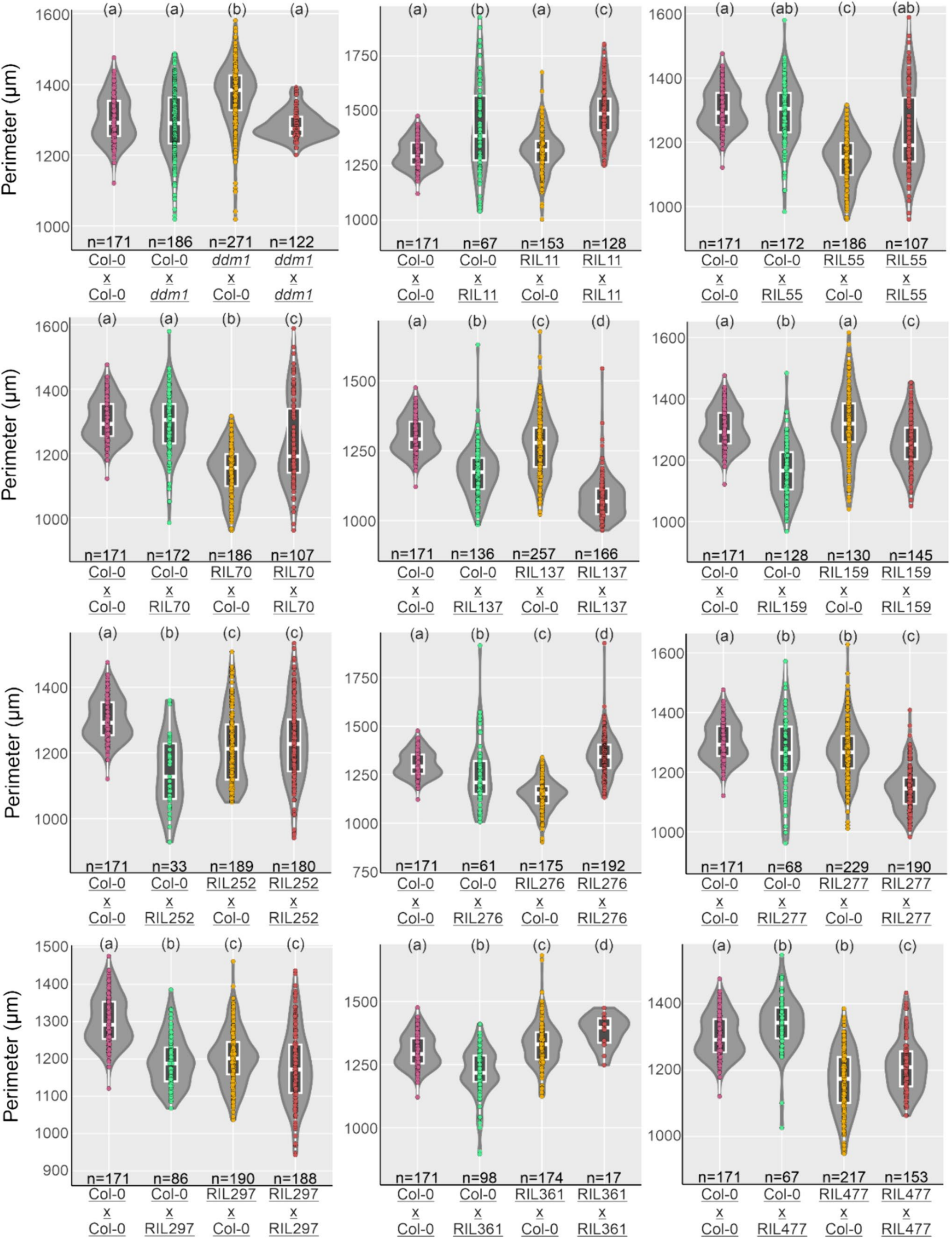
Size of sexual seeds derived from selfed and reciprocal crosses of *ddm1* and best performing epiRILs with the WT. The letters indicate statistical significance for ANOVA (*p* < 0.05)

Finally, we tested whether hypomethylation at MM382 correlated with the size of sexual seeds. This was not the case, as seven of the eleven epiRILs tested produced selfed seeds that were smaller than the WT (Fig. 2). We then performed reciprocal crosses of the selected epiRILs with WT. Sexual seed size in DNA methylation mutants was reported to depend on the parent-of-origin (Xiao et al. 2006): *ddm1* and *met1* used as mothers would lead to large seeds, while the same genotypes used as fathers resulted in small seeds. We confirmed that indeed *ddm1* x WT crosses yield slightly larger seeds, compared to WT x WT crosses (Fig. 2). However, contrary to previous reports, we did not observe a paternal effect of *ddm1* in determining seed size (Fig. 2). Nevertheless, the size of seeds originating from epiRILs X WT crosses did not follow a specific pattern, although there were some parent-of-origin effects for some epiRILs. Therefore, our data suggest that the effect of hypomethylation at MM382 is specific to asexual, and not to sexual seed formation. This means that even sexual species, like Arabidopsis, carry (epi)genetic determinants that can be potentially manipulated to engineer asexual traits, while not compromising general vegetative development, or even the size of sexual seeds.

In conclusion, we showcased the effectiveness of using epiRILs and epiQTL mapping as valuable tools for identifying genomic regions associated with seed traits. Our findings can help pave the way to better understand the pathways necessary to engineer apomixis in crops.

### Author contribution statement

RP and DDF designed the study. RP, SS and DDF performed the experiments. RP analyzed the data. RP and DDF wrote the manuscript, and all authors approved the final version.

## Supplementary Information

Below is the link to the electronic supplementary material.

Supplementary file1 (DOCX 1075 KB)
