# Supplementary material for: An epiQTL underlying asexual seed formation in Arabidopsis
Source: Plant Reprod. 2024 Jun 5;37(4):463–8. doi: 10.1007/s00497-024-00504-y (PMC11511731; doi:10.1007/s00497-024-00504-y)
Supplement: Supplementary file 1 — Supplementary file1 (DOCX 1075 KB) [file 497_2024_504_MOESM1_ESM.docx]

**Supplements**

**Materials and Methods**

### Plant materials and growth conditions

The lines used in this study were previously described as *met1-3* [(Saze et al., 2003)](https://www.zotero.org/google-docs/?rWlrwF)*, ddm1-2* [(Jeddeloh et al., 1999)](https://www.zotero.org/google-docs/?kO0YeI)*,* and as a set of epiRILs [(Johannes et al., 2009)](https://www.zotero.org/google-docs/?ayBUCB). The collection of epiRILs was obtained from the Versailles Arabidopsis Stock Centre, INRA, France (<http://publiclines.versailles.inra.fr/epirils/index>). Primers for genotyping can be found in Table S1.

Seeds were sterilized with 5% commercial bleach with 0.01% Triton X100 for 5 min followed by three times washing with 99.6% Ethanol. The sterile seeds were plated in ½ MS-medium supplemented with 1% sucrose. The plates were kept at 4 °C in the dark for 48 h for stratification. Plates were then transferred to a growth chamber (16/8 h light/dark; 50 μmol.s^−1^.m^−2^; 22 °C). After 10 days, the seedlings were transferred to soil and grown in a growth chamber (16 8 h light/dark; 150 μmol.s^−1^.m^−2^; 22° C; 70 % humidity). The epiRIL autonomous seed phenotypes were assayed in the greenhouse of the University of Potsdam. Other physiological experiments were carried out in growth chambers, as described above.

Physiological assays

To induce autonomous seeds, the hormone treatments used in the experiment contained 0.1% of ethanol, 0.01% of Silwet L-77, and 100 μM of 2,4-Dichlorophenoxyacetic acid (2,4-D). To ensure accuracy in the results, a control Col-0 WT group was also included in all experiments. Two days prior to anthesis, the flowers were emasculated and after two days they were treated with the 2,4-D solution. At the designated time intervals, usually 3 days after treatment (3 DAT), the treated pistils were collected and prepared for microscopy examination.

For root size measurements the seeds were germinated in vertical plates with ½ MS-medium supplemented with 1% sucrose. For auxin treatments the plates were supplemented with 10 nM 2,4-Dichlorophenoxyacetic acid (2,4-D). After eleven days, photos of the plates were taken with a Keyence VHX digital microscope.

For leaf size, the individual leaves of each plant were labeled with a marker pen. At the time of maximum leaf expansion, the 6th and 7th leaf of each plant were collected, glued onto a sheet of paper and scanned using a commercial scanner.

For measurement of mature sexual seeds, the flowers were emasculated and manually crossed two days after. The mature seeds were imaged using a Keyence VHX digital microscope.

### Seed clearing and microscopy

For clearing of ovules and seeds the whole pistils/siliques were fixed with EtOH:acetic acid (9:1), washed for 10 min in 90% EtOH, 10 min in 70% EtOH and cleared overnight in chloralhydrate solution (66.7% chloralhydrate (w/w), 8.3% glycerol (w/w)). The ovules/seeds were observed under differential interference contrast (DIC) optics using a Leica DM2500 microscope (Leica Microsystems).

Seed perimeters and root lengths were measured from microscopy images using Fiji software. The size of mature seeds was determined using the built-in software in the Keyence VHX digital microscope. Leaf size was determined by scanning the leaves on a commercial scanner and measuring using ImageJ. Plots and statistical analyses were done in RStudio.

### Mapping of epiQTLs

Out of the 123 epiRILs belonging to the core collection, we analyzed 116. The remaining 7 RILs either did not germinate or did not flower in our greenhouse conditions. To accommodate the considerable number of plants for this analysis, we conducted the experiment in five different batches, each with its own WT control (Col-0). Due to variations in the size of WT autonomous seeds at 3 DAT between batches, we normalized the measurement data for each epiRIL to the respective WT control in each batch of plants (**Fig. S2**). We proceeded to map QTLs based on the epiRIL data. Shortly, a sample of 25 autonomous seeds was randomly selected for each epiRIL and their size was measured. To ensure comparability, the data was normalized by dividing each data point with the mean of WT based on the corresponding batch of plants. The autonomous seed phenotype was then classified into four classes based on the ratios obtained in each sample, namely Class 1: Ratio < 0.85, Class 2: Ratio 0.86 to 1.00, Class 3: Ratio 1.01 to 1.15 and Class 4: Ratio > 1.16. A scoring method was then generated for counting and transforming the frequencies of each ratio:

$$Score={(F}_{Class1}*1)+{(F}_{Class2}*2)+{(F}_{Class3}*3)+{(F}_{Class4}*4)$$

The score of each epiRIL was used as a trait. Mapping of epiQTLs was performed using the ‘scanone’ function of the R/qtl package for R [(Broman et al., 2003)](https://www.zotero.org/google-docs/?fQmtW1) combining experimental phenotypic data (autonomous seed score) with the recombination map of differentially methylated regions (DMR) generated previously [(Colome-Tatche et al., 2012)](https://www.zotero.org/google-docs/?sdAvJP). A logarithm of odds (LOD) threshold of significance was determined on the basis of 1000 permutations for the dataset (α = 0.05).

**Table S1**: Primers used for genotyping.

| Primer | Genotype | Sequence | Information |
| --- | --- | --- | --- |
| p4_1 | *ddm1-2* | ACGAAGCAACCAAGGAAGAA | Digest with RsaI after PCR:  Mutant - 362 + 205 + 58 + 47 bp  WT - 339 + 205 + 58 + 47 + 21 bp |
| p4_2 | *ddm1-2* | GAGCCATGGGTTTGTGAAACGTA |  |
| p4_3 | *met1* | TAGCCAACAAGTTATCGCTTACTC | WT: (p4_3+p4_4) 700 bp |
| p4_4 | *met1* | TTCGCAAACCATTCTTCACAGAGC | Mut: (p4_3+p4_5) 1000 bp |
| p4_5 | MET1-T-DNA | TAATTGCGTCGAATCTCAGCATCG |  |


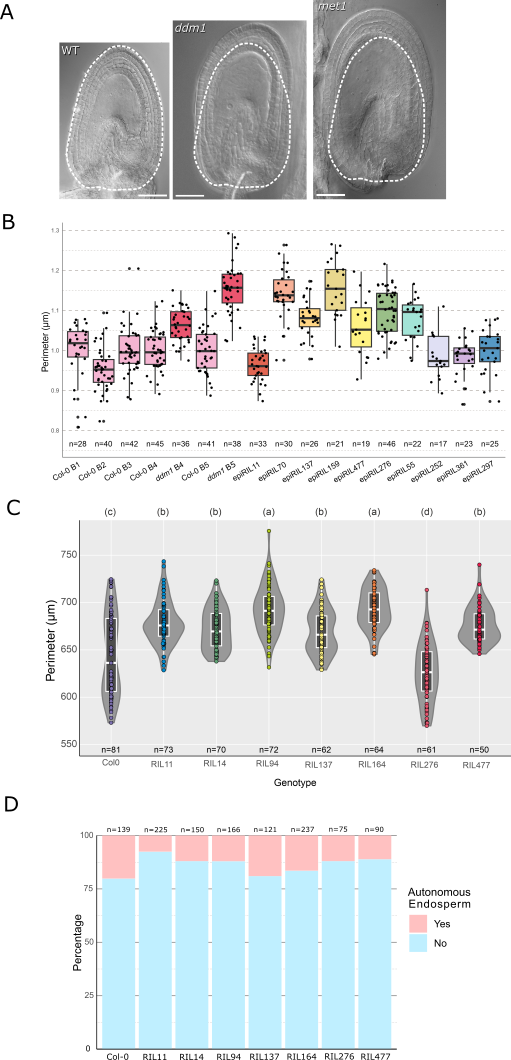


**Figure S1.** (**A**) Autonomous seeds formed after treatment of 100 µM auxin at 3 DAT. Col-0 WT (left) vs *ddm1-2/-* (center) and *met1-3* (right). The white dotted line indicates the size of the WT autonomous seed, to showcase the difference in size between WT and *ddm1*/*met1* phenotypes. The scale bars indicate 50 μm. (**B**) Size of unfertilized ovules of WT, *ddm1-2* and of the 10 best performing epiRILs selected from **Fig. 1**, normalized to the average WT perimeter from each respective batch. The labels B1-5 indicate the batch number of each line. Boxes in pink are WT, red are *ddm1-2* and remaining colors are epiRILs. (**C**) Autonomous seed size at 3 DAT for WT Col-0 and seven epiRILs demethylated in MM382. The letters indicate statistical significance for ANOVA (p<0.05). (**D**) Prevalence of autonomous endosperm after auxin treatments in seven epiRILs demethylated at MM382. The numbers on top indicate the number of ovules/seeds analyzed.


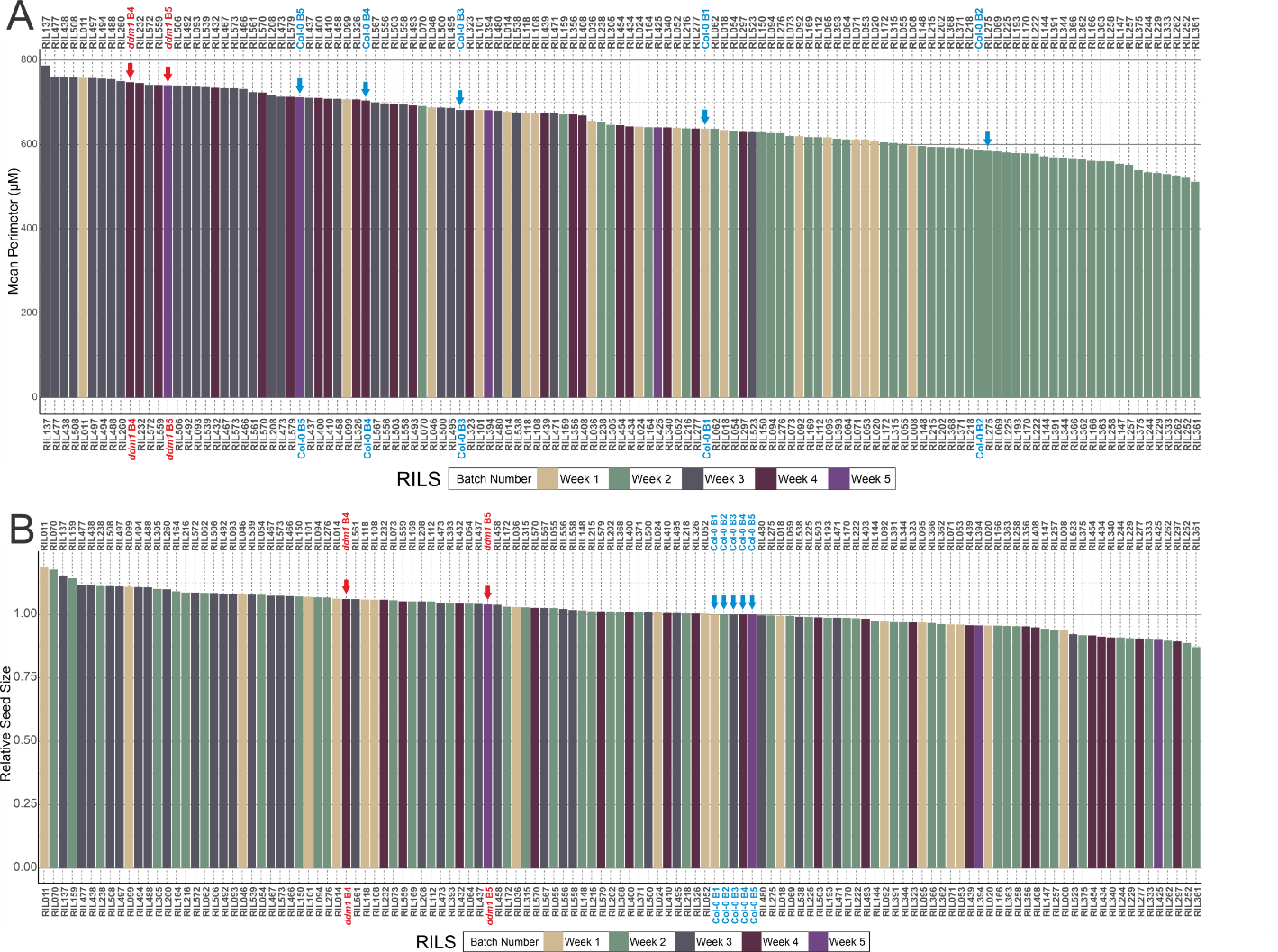


**Figure S2.** Raw Perimeter data (**A**) and Normalized Perimeter data (**B**) of autonomous seed size of the 116 epiRIL lines after 100 µM auxin treatment. Two *ddm1-2* lines and five WT lines are indicated in red and blue label respectively. The graphs are arranged in descending order with decreasing values. The bar color code indicates the batch to which each line belongs to, as detailed in the legend.


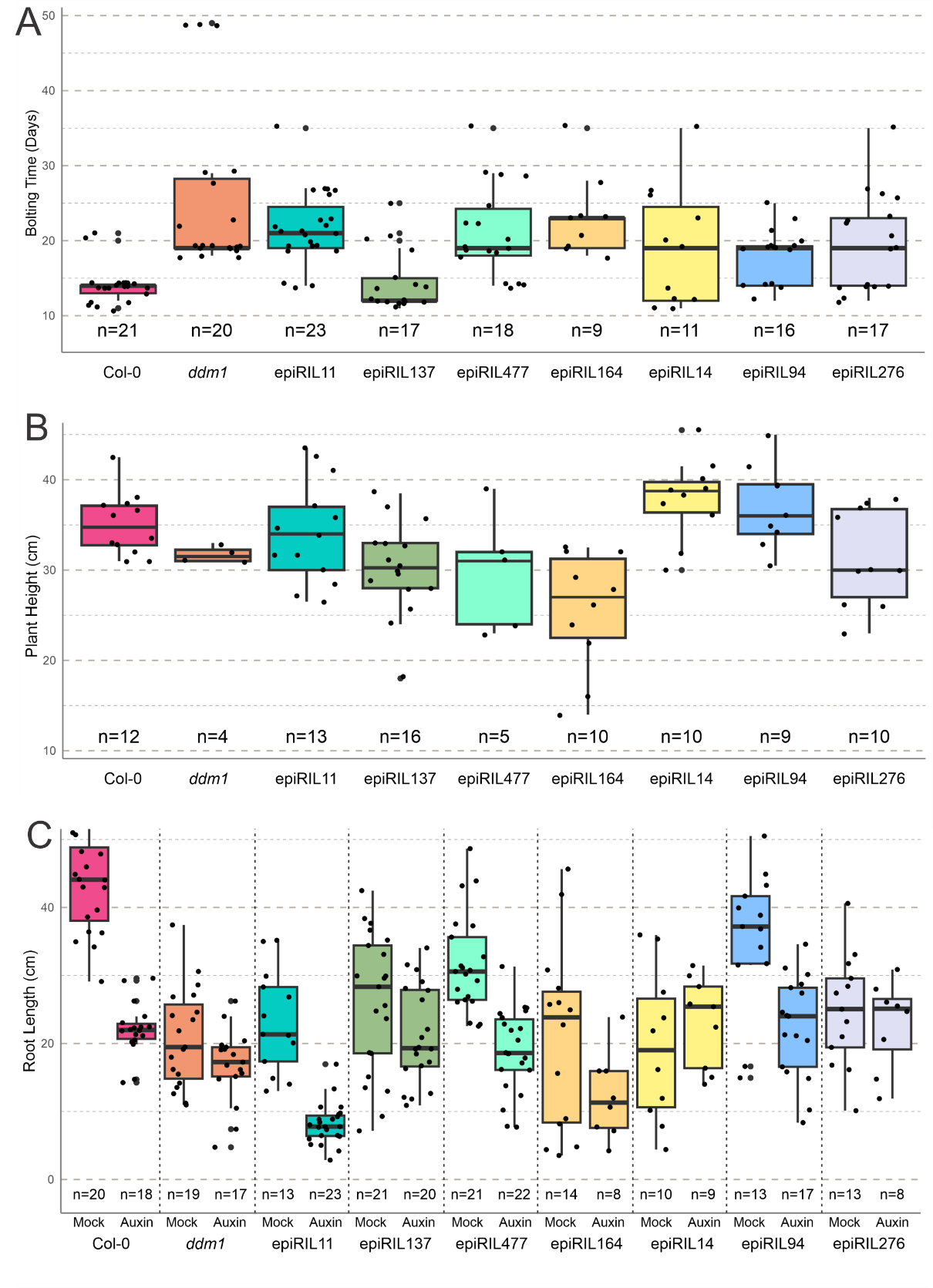


**Figure S3.** (**A-C**) Phenotypic analysis of Col-0, *ddm1* and best performing epiRILs (with hypomethylated MM382) as determined in Fig. 1A. (**A**) Bolting time, (**B**) Maximum plant height and (**C**) Root length, as grown for 11 days in vertical plates in mock conditions (left) and in medium supplemented with 10 nM 2,4-D (right).

**
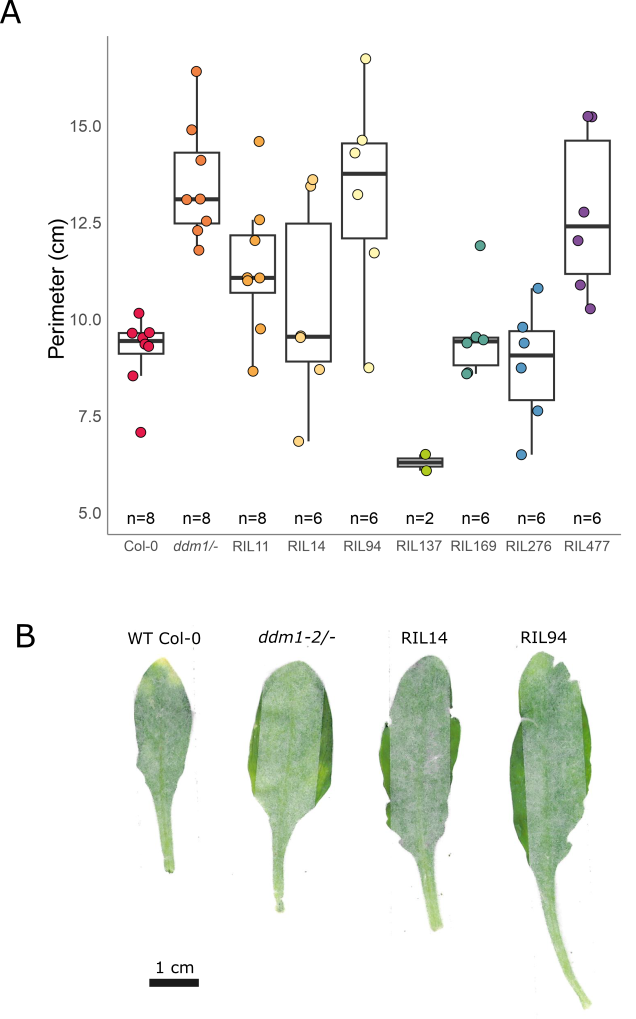
**

**Figure S4.** Leaf size analysis of Col-0, *ddm1* and best performing epiRILs (with hypomethylated MM382) as determined in Fig. 1A. (**A**) Size of fully expanded leaf 6 and 7 in all genotypes. (**B**) Representative images of fully expanded leaf 6 of WT, *ddm1-2* and two epiRILs. Bar indicates 1 cm.

**References for the Supplement**

[**Broman KW, Wu H, Sen Ś, Churchill GA** (2003) R/qtl: QTL mapping in experimental crosses. Bioinformatics **19**: 889–890](https://www.zotero.org/google-docs/?e5scyL)

[**Colome-Tatche M, Cortijo S, Wardenaar R, Morgado L, Lahouze B, Sarazin A, Etcheverry M, Martin A, Feng S, Duvernois-Berthet E, et al** (2012) Features of the Arabidopsis recombination landscape resulting from the combined loss of sequence variation and DNA methylation. Proceedings of the National Academy of Sciences **109**: 16240–16245](https://www.zotero.org/google-docs/?e5scyL)

[**Jeddeloh JA, Stokes TL, Richards EJ** (1999) Maintenance of genomic methylation requires a SWI2/SNF2-like protein. Nat Genet **22**: 94–97](https://www.zotero.org/google-docs/?e5scyL)

[**Johannes F, Porcher E, Teixeira FK, Saliba-Colombani V, Simon M, Agier N, Bulski A, Albuisson J, Heredia F, Audigier P, et al** (2009) Assessing the Impact of Transgenerational Epigenetic Variation on Complex Traits. PLOS Genetics **5**: e1000530](https://www.zotero.org/google-docs/?e5scyL)

[**Saze H, Scheid OM, Paszkowski J** (2003) Maintenance of CpG methylation is essential for epigenetic inheritance during plant gametogenesis. Nature Genetics **34**: 65](https://www.zotero.org/google-docs/?e5scyL)
